# Comparison of vitality states of finishers and withdrawers in trail running: An enactive and phenomenological perspective

**DOI:** 10.1371/journal.pone.0173667

**Published:** 2017-03-10

**Authors:** Nadège Rochat, Denis Hauw, Roberta Antonini Philippe, Fabienne Crettaz von Roten, Ludovic Seifert

**Affiliations:** 1 Institute of Sport Sciences, University of Lausanne, Lausanne, Switzerland; 2 Raidlight-Vertical SAS Outdoor Lab, Saint-Pierre-de-Chartreuse, France; 3 CETAPS Laboratory—EA 3832, Faculty of Sports Sciences, University of Rouen, Rouen, France; Universita degli Studi di Verona, ITALY

## Abstract

Studies on ultra-endurance suggest that during the races, athletes typically experience three vitality states (i.e., preservation, loss, and revival) at the phenomenological level. Nevertheless, how these states contribute to the management and outcome of performance remains unclear. The aim of this study was to determine whether and how the vitality states experienced by runners and their evolution during a trail race can be used to distinguish finishers from withdrawers. From an enactive and phenomenological framework, we processed enactive interviews and blog posts of race narratives. We distinguished units of meaning, which were grouped into sequences of experience; each sequence was then categorized as one of the three vitality states: state of vitality preservation (SVP), state of vitality loss (SVL) or state of vitality revival (SVR). We analyzed the distribution of these vitality states and their temporal organization at the beginning, in the second and third quarters, and at the end of the races, and we qualitatively characterized runners’ adaptations to SVL. Results showed that finishers completed the race in SVP, with overall significantly more sequences in SVP and significantly fewer sequences in SVL than withdrawers. SVR did not discriminate finishers from withdrawers. The temporal organization of the vitality states showed a significant difference in the emergence of SVP from the second quarter of the race, as well as a significant difference in the emergence of SVL from the third quarter of the race. The analysis of adaptations to SVL confirmed that finishers were more capable of exiting SVL by enacting a preservation world when they felt physical or psychological alerts, whereas withdrawers remained in SVL. Our results showed that finishers and withdrawers did not enact the same phenomenological worlds in the race situation, especially in the organization of vitality adaptations and their relationships to difficulties; the cumulative effect of the succession of experienced vitality states differed, as well.

## Introduction

Trail running has become a popular sport over the last 40 years, as noted by Hoffman et al. [1], who observed an increasing number of races per year and more participants per race [2]. Trail running is no longer practiced by a minority of elite runners, but has also become accessible to non-professional runners [3] despite the need for high investment and compromise in terms of training, work schedule and personal life [4]. The races consist of semi-autonomous running along marked trails in natural environments and impose considerable constraints that the runners must adapt to, raising effort, logistic and safety issues. The distances vary between 20 kilometers to more than 300 kilometers, with ultra-trail races generally more than 80 kilometers. During these races, runners risk extreme fatigue and/or exhaustion and at times exceed their personal limits [4]. Ultra-trail racing has therefore been considered an extreme sport or even a dangerous activity [5].

There are multiple ways to analyze this type of ultra-endurance performance. A current trend is the “third-person approach” to identify the determinants of performance. In this case, studies are based on the assumption that performance is dependent on two types of factors: (a) before-the-race factors, which include, for example, training habits [6], the impact of training characteristics on running-related injuries [7], and physical, mental and tactical preparation [4], and (b) during-the-race factors, which include sleep-deprivation effects [8] and neuromuscular damage [9]. Other determinants of performance have been examined by isolating specific characteristics, without distinguishing between these two types of timed factors; these include mood states [10], cognitive functioning [11], personality traits [12], emotions [13], sarcomere disruption [14], and alteration of jump height mechanics after a mountain footrace [15]. However, these approaches partition the unity of runners’ activity into specific processes, which precludes the possibility of understanding runners from a holistic perspective ‒ that is, as capable of compensating a performance deficit in one phase of the race by heightened performance in another phase, or compensating one process by another, such as psychological coping with physiological problems [16]. Another trend in cognitive science has developed in this direction and consists of investigating the way people integrate physiological and psychological factors into a mental unity that emerges at the psycho-phenomenological level [17,18]. From this perspective, human activity is (a) Embedded in the whole dynamics of the changing situation [19], (b) Extended by tools or cultural artifacts (e.g., [20]), (c) Embodied as recurrent sensorimotor patterns of perception and action [21,22], and (d) Enacted by bringing forth a cognitive being’s world with specific asymmetrical relationships between the person and his/her environment [17,23]. This last element of the four-E approach assumes that the cognitive being’s world–whatever that being is able to experience, know, or practically handle–is a constitutive part of human activity that should be investigated in a rigorous phenomenological manner [21]. Phenomenology benefits from a philosophical background and has shown its practical applications in research in the cognitive sciences [24]. The objectives of the phenomenological approach are to analyze experience and describe a phenomenon in terms of how it emerges at the level of consciousness [25]. This approach thus operates at the interface of conscious and unconscious processes, or at the “fringe of consciousness” [26]. Furthermore, by analyzing experience in context, phenomenology roots its analysis in the pre-reflectively experienced lived body [27]. At this level of analysis, accounting for how a person feels and acts in a given situation requires a “first-person approach” (e.g., [24,25]) to identify how experience includes physical events and the synthesis of sensorial events. The heterogeneity of these events thus emerges at the level of mental or cognitive unity or the “feeling of what is happening” [21,28,29]. This *experience* is the sense (including bodily, emotional, cognitive, action and situational dimensions) that corresponds to the manner by which people make worlds emerge, in which they have being and act [30]. Therefore, Di Paolo et al. [31] suggested that a cognitive being’s world consists of *sense-making* instead of information processing, which is key to the computationalist view. Cognition can thus be conceived as a form of embodied action in which a person creates meaning by interacting autonomously with the environment, leading to fluctuations in experience. By rooting the analysis of these fluctuations in the enactive paradigm, which encompasses the dimensions of experience, meaning and asymmetrical interactions, we argue that it is possible to obtain the temporal organization of the salient phenomenological states that characterize experience.

### A multiplicity of phenomenological states in trail running

Based on the four-E approach, we postulated that trail running in a competitive context would shape runners’ activity and experience in such a way that we would be able to identify the emergence of typical phenomenological states. Our challenge was to define the nature of these states, which are singular, context-dependent and peculiar to each runner; therefore, we assumed there would be many conceivable phenomenological states. However, experiences in trail running also have many similarities, as shown in a study about the story of withdrawals [32], which identified the categories that characterized 20 trail runners’ enacted worlds from the start of the race until the moment they decided to quit it. The findings opened new possibilities for analyzing trail running experience by suggesting that the runners went through various phenomenological states that could be clearly labeled in categories. In the same vein, qualitative studies of ultra-endurance runners identified three major states in finishers related to effort management. The first concerned the stressors that impact their experience (i.e., cramping and injuries, gastrointestinal problems, and thoughts about quitting) [16] and characterize a state of suffering. The second concerned the protective processes aiming at preserving oneself, such as psychological coping strategies like setting short-term goals, pace monitoring, hydration and nutrition, and social support [16]. The third state concerned positive emotions, such as group cohesiveness during a part of the race, self-awareness or mental stamina emerging during effort [33], and positive self-talk [4] to revive vitality during the race.

Interestingly, these states correspond to the findings of other targeted studies in physiology that had to do with the state of suffering. For example, sleep deprivation was reported to negatively impact runners’ cognitive performances (i.e., decreased psychomotor vigilance, increased reaction time lapses, inability to stay awake, and sometimes visual hallucinations) [8]. Moreover, long-distance effort was found to lead to emotional disturbances and negative energetic balances [13]. In contrast, physiological evidence also indicated that runners can prevent this state by using anticipatory processes that ensure a form of self-preservation: indeed, less neuromuscular fatigue, muscle damage and inflammation were reported in a 330-kilometer race than in shorter races [9]. According to the authors, the runners adopted a protective pacing strategy during the first half of the race, which reduced muscle damage.

Hence, these studies have all shown that the experience of running a trail race corresponds to the various dimensions of the runners’ activity [34]. These dimensions document the processes involved when runners prepare for a race or confront difficulties in the race, and during key moments of performance. Moreover, the notion of vitality embeds all these dimensions. Subjective vitality is “a conscious experience of possessing energy and aliveness” ([35], p.530), which can also be absent in other contexts. These authors emphasized that vitality is a psychological experience that depends on a physiological state (e.g., fatigue, illness) and psychological states. Therefore, based on the evidence that vitality has phenomenological anchorage, we assume that it (2) embeds physiological and psychological processes that constitute heterogeneous information synthesized into mental unity and (2) can fluctuate according to the context.

There are several ways to analyze vitality, which has been conceived as a global state [35] and has been used to investigate well-being and mindfulness [36,37] with a validated scale to measure it [38]. However, although this scale provides information about a subject’s general well-being in relation to other factors (i.e., friendship, intrinsic values), the temporal fluctuations of vitality still remain unclear. We postulate that trail runners’ experiences and meaning are timely and structured [39], reflecting an *emergent* process of experiencing the race situation. Indeed, as previously emphasized, runners experience phenomenological vitality states, and analysis of the temporal organization of these states might provide an understanding of how they emerge. This in turn might provide insight into the runners’ adaptations to the phenomenological vitality states and thereby explain race outcomes (finish vs. withdraw).

We sought to connect this conception of vitality with the existing literature about trail running and endurance running and identified three vitality states. The first was a “state of vitality loss” (i.e., SVL) that runners may experience during a trail race, prompting them to withdraw because of the constraints typical of this sport [40]. Conversely, runners may also live a “state of vitality revival” (i.e., SVR) in which they feel good sensations and positive moments. Third, the protective processes emerging during these races suggest that runners experience a “state of vitality preservation” (i.e., SVP), which suggests that withdrawers might not be exclusively those who experience SVL, but also those who cannot preserve themselves sufficiently. They therefore experience more vitality loss during the race and progressively become unable to finish [32]. Comparing how these phenomenological vitality states evolve in finishers and withdrawers may provide insight into the processes that lead to withdrawal from or completion of the race. By providing a qualitative description of the race outcome, the temporal organization of SVL, SVP and SVR may be important not only to understand how an immediate state can impact the following states, but also to further explore how the positive mood states that runners experience during long races are able to re-launch them completely after a period of suffering or vitality loss. We hypothesized that (1) performance outcomes (finish vs. withdraw) would result from the temporal organization and interactions of these phenomenological states and (2) the lack of a vitality revival during a race would prompt a runner’s decision to withdraw.

To summarize, few studies have investigated long-distance running outcomes (finish vs. withdraw) and the phenomenological vitality states that have been observed during the races, which include physiological, emotional and cognitive processes. We report here the results of an investigation of the phenomenological vitality states during multiples races to understand why runners finish or withdraw from a race. We used an enactive and phenomenological perspective to characterize the distribution and temporal organization of these states and runners’ adaptations to them.

## Material and methods

### Research design

For the analysis of experience, the sport and psychological sciences have mainly used two approaches to consider its temporal organization [41,42]. Within an enactive framework, the “course of experience” analyzes experience through the succession of enactments at the level of what agents are able to perceive, feel, know and do [43,44]. Here, sense-making is studied by identifying the succession of linkages between action and situation considered at the level of what is meaningful for an agent, using a semiotic approach to cognition and action inspired by Peirce (1931–1935) [45–48]. The course of experience reflects the world enacted by genuine agents in situation through the characterization of elementary units of meaning (EUMs) that mainly emerge from the association of the agent’s intentional state (i.e., the field of possible actions he or she can undertake) and situation-related judgments of a proprioceptive, perceptive or memory-based nature (i.e., representamens) [43,49]. The course of experience is thus the chaining of these EUMs during a period of an agent’s activity characterized by emergent or higher-order structures of meaning, such as sequences (i.e., the succession of EUMs that corresponds to a similar agent’s concern). A key element that distinguishes the course of experience from the narrative approach is the use of second-person reports coupled with third-person descriptions, such as video recordings or biomechanical data [47]. Second-person reports are collected through a process by which traces of past activity are presented to the agent to stimulate a re-enactment [50–52]. Here, agents are invited to re-experience and describe the stream of past experience in relation to these traces by adopting a stance that consists of reliving their own past experience, although deliberately ignoring the outcome [52]. In doing so, they re-enact meaningful parts of their past experience. The confrontation with each trace of the periods and shifts in an agent’s experience is considered as a new situation, although the new meaningful experience that is built has many similarities with the one that the agent lived in the past [46,49]. As this approach has been successfully used in studies analyzing performance outcomes in various sports (e.g., [51,53–55]), we assumed it would be suitable for trail running.

The second approach, narrative inquiry, has received a great deal of attention in relation to the report of the stream of activity. Bruner [56], for example, suggested that our personal knowledge and experience are organized through narratives of sequences of events that correspond to a psycho-phenomenological level. According to Gibbs [22], agents describe those events that are meaningful for them through narration. The narrative structure provides landmarks that meaningfully and in timely fashion discretize the stream of the events that form the structure of a story of a person’s experience [57]. For Bruner [56], Propp [58] and Greimas [59], the analysis of narratives shows that their structure possesses properties that represent how the meaning of sequences of events is experienced (e.g., the existence of a plot, obstacles to overcome, problems to solve, presence of allies, the necessity of having continuity in one’s identity, the possibility of expressing mood or the search for meaning). Relatedly, researchers have shown increasing interest in experience-sharing blogs and forums as an innovative tool for analyzing narratives. Blogs provide a space for personal expression, and Bortree [60] observed that teenage girls were more likely to express their thoughts and report on their daily activity by recounting their experiences on their blogs. Blogs are thus suited for gathering experiential data because these are expression spaces in which people feel comfortable to talk about their personal experiences [61]. The accounts posted on blogs can thus help us obtain valuable narratives of the personal experience that has marked runners [62,63], as a participatory sense-making process emerges from the interaction between the teller and the reader [64]. Sport sciences have also shown great interest in these narratives in the fields of health (e.g., [65]), physical activity and leisure [66], physical education (e.g., [67]), adapted physical activity [68] and elite sport (e.g., [69]).

By putting together the course-of-experience and narrative approaches, runners’ reports of experience have provided insight into the way temporal organization reflects the segmentation of separate phenomenological states [70–73], including the key shifts in the vitality states during a trail running race. These successions of t-time vitality states enacted by the athletes emerged at the phenomenological level as “perceptual packets” [39,74] forming sequences (e.g., SVP, SVL, SVR) with unpredictable duration and chaining. They characterized runners’ step-by-step experiences that depicted their singular story of vitality states during a race.

Thus, our research was designed to process two types of data: (a) recorded and transcribed commentaries elicited by researchers during enactive interviews (EIs) with athletes who were confronted with traces of their own past activity and asked to rebuild their experience (i.e., EI data) and (b) freely written accounts of races retrieved from blogs published on the Raidlight community website (i.e., blog data).

### EI data processing

#### EI participants

Thirteen French runners (nine males and four females) who participated in the “Grand Raid de la Réunion” volunteered to participate in the study. All were between 26 and 52 years old. They were recruited (a) through a message posted on the Raidlight community forum or (b) by responding to a notice before the race. Snowball sampling enabled us to obtain these participants. The protocol was carried out during a trail running event on Reunion Island, which comprised three races in which our sample participated, according to the following repartition (Table 1):

**Table 1:** Repartition of the EI participants (N = 13).

Race name	Length (km)	Positive elevation gain (m)	Finishers (n)	Withdrawers (n)
**“Diagonale des Fous”**	173	9996	3	5
**“The Bourbon Trail”**	97	5655	2	2
**“The Mascareignes Trail”**	65	3922	1	0

During this event, the race statistics indicated a 48.1% rate of withdrawal. Participants were between 24 and 74 years old (mean age = 43 years old) [75] and 90% were men and 10% were women. The six finishers in our sample were ranked between 8^th^ place and 1130^th^ place. Therefore, the proportion of finishers in our sample matched the race statistics, and the demographic characteristics suggest that the runners in our sample were representative of the diversity among the participants.

#### EI data collection

EI data were collected using traces of the runners’ past activity. The traces were two maps of the race: the first provided information about aid stations and geographic landmarks (depicting the view of the route from above) and the second showed the elevation changes along the route.

EIs took place shortly after the race, lasted between 60 to 120 minutes, and were recorded. During the EIs, the runners were confronted with these traces. The interviews were designed to provoke the re-emergence of elements of past experience when the participant was bodily face to face with traces of his/her own activity. The runners were asked to show, tell about and comment on their experience. In doing so, they revealed how they handled it online by building new meanings (i.e., re-enactment) or activating pre-existing ones (i.e., remembering) [47,48,53,76]. The researchers took steps to prevent the runners from retrospectively recalling their experience. First, they asked the runners to avoid judging their activity (i.e., judgment suspension) and to concentrate on explaining the experience, as suggested for phenomenological research [77]. Second, the traces of past activity presented during the EI were aimed at stimulating the runners to re-enact the stream of their experience in situation while deliberately ignoring the outcome [51]. Third, to ensure that the runners were not retrospectively recalling their race experience, the researcher took careful note during the interviews that all runners, whatever their outcome, related positive and/or negative experiences, such as pain, joy, ease, etc. In addition, if a runner emphasized a positive account during the interview, the principle of in-depth qualitative research dictated that the researchers looked for a more accurate and authentic report of experience, always in relation to the unfolding situation.

Verbal prompts were used to elicit further information about the meaning of each runner’s activity, including sense-making from an enactive perspective, this being the actions inseparably coupled with their experience and following their own story of the race: involvements, units of meaning (i.e., action) and representamens, as has been done in previous research using the course-of-experience approach [44,45,47].

The involvement (I) refers to the possibilities that are conceivable by the actor in the situation; it expresses how he/she enters into activity (e.g. “What were you concerned about at this moment?”). The unit of meaning (UM) depicts the breaks of the runner’s race story that correspond to the fraction of activity that is meaningful for him/her at each moment (e.g. “What were you doing?”). The representamen (R) corresponds to what the runner is feeling in relation to the UM or the feeling of what is happening in the unfolding situation (e.g. “What was significant for you at this moment?”).

#### EI data coding

The data treatment was grounded in a phenomenological method that articulates the inductive and deductive approaches by identifying the descriptions of a phenomenon that can be clustered into discrete categories and then put together to identify the core and the structure of the experience (see Starks & Brown Trinidad, p. 1373 [77]).

Hence, the phenomenological data were inductively coded into units of meaning, then deductively classified using first categories of meaning (i.e., involvements, representamens, and units of meaning), and last classified as one of the three vitality states identified in our literature review (i.e., SVL, SVP and SVR). This approach has been used in various studies in sport science, ergonomic and educational research [78–80]. It required a succession of four data coding steps.

First, the enactive interviews were transcribed verbatim. Second, a general coding system for describing the settings of activity was established for each runner (Table 2). The system put together all the information collected in the EI with traces of past activity; this allowed us to rebuild the story of the race as experienced by each runner.

Example: Extract from an EI

“*Researcher: Please comment on your race as you lived it, tell me when there were changes. You have the race maps to help you.*

*Runner:* *The atmosphere was great this year! There were people everywhere, enthusiastic at the start and this enthusiasm lasted a really long time. The weather was very good, warm, so I started with a T-shirt; just a T-shirt so it was great, encouraged by the crowd, by people on the sides of the trails. Then we started, I didn’t want to start too fast, because I was recently injured, so I started to run at a good pace but I moderated it in order to preserve myself.*

*Researcher:* *Did you feel pain?*

*Runner:* *A little bit. But… It was not… Actually I injured my adductors in July. I had more or less recovered with physiotherapy and I knew that I shouldn’t start too fast to avoid getting hurt. However, in the meantime, I had sciatica so these last few days, it was painful and I told myself: the sciatica will pass because often before the start I feel pain everywhere… I’m experienced with that but I started confident anyway, so I’ll see, I’ll go as far as I can and it’ll be a tough race as usual, so at the first aid station, it was perfect, I drank water, I needed nothing and I continued in the direction of Berive, many people were outside and it was very motivating to see people everywhere. I really enjoyed it.”*

**Table 2:** Example of UM coding system from EI data.

**Unit of meaning (UM)**	Starts the race in St-Pierre wearing just a T-shirt	Runs at a good pace but moderates her speed	Drinks water at the first aid station	Continues to Berive
**Involvement (I)**	Shouldn’t start too fast and is confident	Shouldn’t start too fast	-	Motivated
**Representamen (R)**	Great atmosphere with the crowd at the start/ sciatica pain	People on the sides encouraging	It’s perfect, no need of anything	Many people encouraging

Third, these UMs were grouped into sequences that referred to the same story during a part of the race. Two UMs belong to the sequence “if one is partly determined by the outcome of the other or if they both refer to the same theme” [81]; their formulation synthesized the content of the UM (Table 3).

**Table 3:** Example of sequences identified from the UM coding.

**Unit of meaning (UM)**	Runs on a unknown trail segment	Crosses a village	Leaves the village	Keeps on crossing villages	Finds a known path	Arrives at the aid station
**Involvement (I)**	Destabilized because he is on a unknown path	Angry	Tired, bad mood, less concentration	Angry	Focused on the race again	Can stick to his plans again
**Representamen (R)**	Feeling of loss of control, negative emotion	Technical difficulties, negative emotion	-	It’s hard	Many people are encouraging	Members of his support team, feeling good
**Sequences**	Runs angry and less concentrated on an unknown trail segment				Runs relaxed, with good sensations on a known path	

Fourth, each sequence was classified into one of the three types of vitality states identified in the coding system, according to the following classification (Table 4). To identify the states, we collected all the sequences and examined their content (i.e., involvements, representamens and units of meaning). By doing so, we were able to classify them in their corresponding vitality state. For greater clarity, we normalized the formulation of the content of each category succinctly to portray all the typical dimensions of the trail runners’ experience.

**Table 4:** Criteria for coding the sequences as phenomenological vitality states.

	State of vitality revival (SVR)	State of vitality preservation (SVP)	States of vitality loss (SVL)
**Involvement (I)**	Lead the race, get ahead of competitors, motivated to overcome, gain time or increase advance	Be careful with the pace, preserve oneself, energy, keep physical integrity, do not get hurt	Hold on, struggling to go on
**Unit of meaning (UM)**	Run/walk fast, accelerate, decide not to stop at an aid station, pass other runners	Slow down, do medical procedure, use logistical supports, force oneself to stay at a perceived slow pace, deliberately do not pass a competitor, take breaks, hydrate, eat, sleep	Constrained activity such as slow down, walk slowly, lose the route
**Representamen (R)**	Other runners’ activity, feeling of having much energy, speed is higher than expected	Feeling of ease, pleasure	Bad sensations, difficulty, pain, tiredness, cold, negative emotions, bad sleep, hallucinations, concerns about not being able to finish the race, feeling of going slower than expected, people passing, thoughts about abandoning

**Fig 1:**
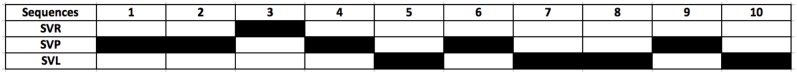
Succession of the vitality states in sequences identified from the runners’ courses of experience.

Thus, for each athlete we obtained a succession of the vitality states identified in their course of experience (Fig 1). As shown in this figure, the same vitality state could be distinguished in two successive sequences when, for example, the involvement and the representamen changed focus while the general theme stayed the same. To ensure the validity of the data coding, the steps of data treatment were carried out independently by three researchers who then compared their respective codings in order to find common agreement.

### Blog data processing

#### Blog data selection

Thirty-three blog posts on the community website of the Raidlight brand were selected from among several types of online contents. All were post-race accounts of experience. We collected 17 blog posts reporting finishing the race and 16 reporting withdrawal. The data collection complied with the terms and services of the Raidlight website.

#### Blog data collection

In order to make the narrative contents compatible with the course-of-experience analysis, we searched each of them for the same information on meaning as for the EI data collection. When the blog data were not suitable for this coding system, they were deleted from our database (n = 5, 2 finishers and 3 withdrawers). The selection targeted trail running experience in various races (M = 94.90 km, SD = 39.92) according to the following repartition (Table 5). In total the data set was composed of 28 blog posts (15 finishers and 13 withdrawers).

**Table 5:** Repartition of the blog data participants (N = 28).

Race name	Length (km)	Positive elevation gain (m)	Finishers (n)	Withdrawers (n)
**“CCC”**	106	6100	8	1
**“UTMB”**	170	10000	0	3
**“Nicolet-Revard”**	51	2700	6	0
**“TransjuraTrail”**	72	3200	1	1
**“UTPMA”**	105	5600	0	2
**“GRP”**	80	5090	0	1
**“Infernal des Vosges”**	160	7300	0	1
**“TVS”**	110	8375	0	1
**“Ecotrail”**	50	3681	0	1
**“TGV”**	73	3800	0	1
**“Saintélyon”**	72	1950	0	1

#### Blog data coding

As we obtained the same type of data as in the EIs, we applied the same coding procedures. We successively established the general coding system for each blog and the corresponding vitality states coding system (Table 6).

“At 8 p.m. at St-Pierre, I’m among the first ten runners to enter the start area. I feel stress and stamina, mixed with the feeling of living something exceptional. I feel a bit nervous because I arrived by plane yesterday and I’m afraid I lack sleep. Anyway, I’m here with one single idea: finish. At 11 p.m., I’m literally transported by the stream of 2182 runners behind me. With D. we’re starting fast as planned. Too fast, sometimes at 14 kilometers per hour in the first 7 kilometers, and we passed the first checkpoint in 40th place. We start the ascent. D. slows down and around the 12th kilometer, I start chatting with A., a runner I met in another race. This makes make realize I should not be here, and even if I feel good, I slow down.”

**Table 6:** Example of coding system for blog data.

**Unit of meaning (UM)**	Enters the start area	Runs the first 7 kilometers fast with D	Passes the first checkpoint	Starts the ascent chatting with A	Realizes he is running too fast	Slows down
**Involvement (I)**	Wants to finish the race	Planned to start fast	-	-	-	Should slow down in spite of his good sensations
**Representamen (R)**	Feels stress, nervousness and fear of lacking sleep	Reaches a speed of 14 kilometers per hour	Holding the 40^th^ place	His friend slows down	Feels good	Feels good

#### Ensuring data validity

Several measures were taken to ensure the comparability of the data. First, two investigators, each experienced at conducting qualitative research independently, coded the 41 data transcripts according to the criteria for the general and vitality states coding systems. An agreement rate of 90% was obtained between the two coders. A third coding session was conducted to reach consensus for the 10% disagreement.

Second, the number of UMs collected with EIs and blogs were compared to statistically assess whether they were of the same order of size. We hypothesized that a non-significant difference would reflect a comparable segmentation of the courses of experience. This would confirm the agreement across reports on the criteria that the runners used to indicate meaningful breakpoints in their experience for a comparable segmentation of narratives. A chi-square test compared the repartition of the three vitality states (i.e., SVR, SVP, SVL) between the two datasets (i.e., EI and blog coding) and indicated a non-significant difference (χ^2^(2) = 0.301, p = 0.860), suggesting that the number of sequences in SVR, SVP and SVL did not significantly differ when coding blog and EI data.

In addition, we compared the number of sequences between the two datasets and found no significant difference (Welch’s test of two independent samples with inequality of variances: t(16.257) = 1.233, p = 0.235), and we compared the length of the races between the two datasets and found no significant difference (Welch test: t(14.455) = -1.877, p = 0.081).

Statistical results on the assessment of the comparability of the EI and blog data authorized us to gather them into a single dataset for the next step of data processing.

### Ethics statement

The protocol was approved by the ethics committees of both the University of Rouen and the University of Lausanne (joint agreement) and followed the guidelines of the Declaration of Helsinki. Procedures were explained to the participants, who then gave their written informed consent to participate.

### EI and blog data processing

We performed a logistic regression to explain the dichotomous outcome (finish vs. withdraw) with two independent variables: the number of kilometers of the race and the number of sequences. The results indicated that the number of kilometers was significant (exp(B) = 1.048, p = 0.013: when the number of kilometers of the race increased, the chances of finishing it increased as well) but not the number of sequences (exp(B) = 0.942, p = 0.540). Therefore, we used percentages instead of counts in the subsequent analyses.

The data were processed in four steps to determine whether the race outcome (finish vs. withdraw) could be characterized by: (a) the distribution of the vitality states, (b) their temporal organization, (c) the runners’ immediate adaptation to the experienced state of vitality loss and (d) the contents of the runners’ adaptations.

#### Distribution of the vitality states

The distribution of the vitality states in relation to the race outcome (i.e., finish vs. withdraw) was determined by comparing the means and standard deviations of the percentages of each vitality state for finishers and withdrawers. T-tests compared the repartition of SVR, SVP and SVL in finishers and withdrawers; when variances differed between the groups, we used the Welch test. Normality was tested with the Shapiro-Wilk test. All tests were performed using the significance level of 5% (p≤0.05) with SPSS statistical software. Furthermore, for each course of experience, we quantified the total number of sequences of experience and their repartition in the vitality states of four periods: the beginning, the second quarter, the third quarter and the end of the race.

#### Temporal organization of the vitality states

The temporal organization of the vitality states in relation to the race outcome was determined using measures or cumulative measures. First, we analyzed the number of SVL and SVP sequences in the four race periods to detect a difference in pattern between finishers and withdrawers. Moreover, we performed a logistic regression of eight independent variables (four measures of SVL and SVP), which were important to predict race outcome via an iterative method (forward, likelihood ratio). Second, we divided the cumulative number of sequences per category of vitality state by the cumulated number of sequences in the four race periods as explained above. To do so, we identified the relative accumulation of each vitality state for each sequence of experience (Table 7).

**Table 7:** Example of emergence of vitality states for each sequence.

Sequences	1	2	3	4	5	6	7	8	9	10
**Course of vitality states**	SVP	SVP	SVR	SVP	SVL	SVP	SVL	SVL	SVP	SVL
**Cumulative number of SVR**	0	0	1	1	1	1	1	1	1	1
**Cumulative number of SVP**	1	2	2	3	3	4	4	4	5	5
**Cumulative number of SVL**	0	0	0	0	1	2	3	3	3	4

Therefore, we calculated the ratio of each vitality state for each sequence: for instance, the ratio of SVP at the 6^th^ sequence was 4/6 = 0.66. For each vitality state and for each race, we calculated the ratio at the one-third and two-third points and the end of the race.

Thus, each course of experience was split into four periods in which we obtained the percentage of the state of vitality experienced for each state in each sequence and the percentage of each cumulated state each period. T-tests compared the percentages of cumulated states in the four periods by controlling type I error, i.e., using a p-value of 0.0125 (i.e., 0.05/4) as the level of significance (Bonferroni approach). As before, we checked the homogeneity of variances and normality.

#### Runners’ immediate adaptation to the state of vitality loss

The runners’ adaptations after experiencing a state of vitality loss during the race were assessed to determine whether finishers and withdrawers could be distinguished by their ability to reorganize their activity when they went through various vitality states. To do so, we calculated the types and frequency of vitality states at t+1 after a sequence in SVL for finishers and withdrawers. A chi-square test compared the number of sequences in SVR, SVP or SVL after a sequence of SVL.

#### Content of the runners’ adaptations

The analysis of the content of the runners’ adaptations compared the representamens and involvements between finishers and withdrawers in the sequences in SVL and SVP, based on the following assumptions: (a) the more time runners spend in SVL, the more probable it is that they will withdraw, (b) the more time runners spend in SVP, the more probable it is that they will finish, (c) attempts to cope with SVL will help runners exit from this state of vitality loss, and (d) being able to maintain SVP will help runners to experience less SVL. We clustered these two elements of meaning contrasting finishers and withdrawers into types using thematic analysis, as suggested by Vaismoradi [82]. Our twofold intent was to determine whether during the SVL sequences runners were only in a state of suffering or were also trying to enact a new experience in response to difficulties, and whether during SVP sequences they were only running without being aware of this preservation state or were actively trying to maintain this state.

## Results

### Distribution of the vitality states

The repartition of the sequences in each vitality state revealed that finishers had significantly more sequences in SVP than withdrawers (i.e., 59.5% units of preservation for finishers whereas withdrawers had 39.8% units of preservation, t(39) = 6.782, p = 0.000). Moreover, finishers had significantly fewer units of SVL than withdrawers (18.7% for finishers and 42.2% for withdrawers, t(39) = -7.853, p = 0.000). There was no difference in the units of vitality revival (SVR) between finishers and withdrawers (t(39) = 1.279, p = 0.208) (Table 8).

**Table 8:** Percentages of the three categories of vitality states in blogs and EIs (N = 41).

	SVR		SVP		SVL	
	Finishers	Withdrawers	Finishers	Withdrawers	Finishers	Withdrawers
***M***	21.74	17.98	59.51	39.81	18.75	42.21
***SD***	8.61	10.19	8.20	10.33	8.20	10.80

### Temporal organization of the vitality states

**Fig 2:**
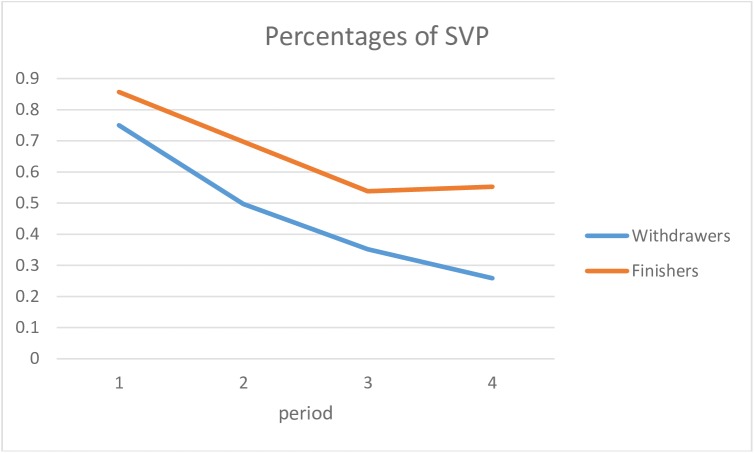
Estimated means of sequences in SVP in finishers and withdrawers in the four periods.

The evolution of sequences in SVP in the four periods of the race for finishers and withdrawers is represented in Fig 2. Throughout the race, the two groups increasingly diverged, although both followed a similar pattern of decrease.

**Fig 3:**
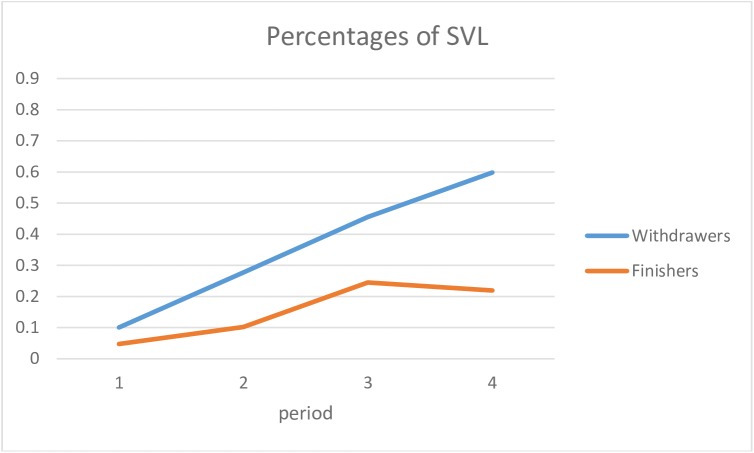
Estimated means of sequences in SVL in finishers and withdrawers in the four periods.

For the SVL category (Fig 3), the evolution was also different for finishers and withdrawers, following a similar pattern of increase for the first three periods, but different trajectories for the last period.

The cumulated frequency of the states in each period (i.e., beginning, second quarter, third quarter, end of the race) while controlling type I error (i.e., Bonferroni approach with level of significance of 0.05/4 = 0.0125) showed the following:

- For SVP: There was no significant difference in the beginning between the two groups (t(39) = 0.852 and p = 0.400). Then in the second quarter a significant difference was observed (t(27.453) = 2.783 and p = 0.011), which remained in the third quarter and the end of the race (resp. t(39) = 4.756 and p = 0.000, t(39) = 6.782 and p = 0.000).
- For SVL: There was no significant difference in the beginning (t(39) = -0.631 and p = 0.532) and in the second quarter (t(30.076) = -2.597 and p = 0.014). A significant difference was observed in the third quarter (t(39) = -5.050 and p = 0.000) and the end of the race (t(39) = -7.853 and p = 0.000).

A logistic regression assessed the temporal organization of the SVL and SVP states simultaneously. The iterative procedure of the method designated the following as the two most important measures to explain the race outcome: the measure of SVL at the end and the measure of SVP in the second quarter (Table 9). With these two measures, we could accurately predict 95.1% of the runners’ outcomes.

**Table 9:** Results of the logistic regression to explain the race outcome (i.e., finish or withdraw).

	A	Wald statistics	p
SVL end	-15.15	6.619	0.010
SVP second quarter	9.52	6.341	0.012

A higher number of SVL sequences at the end decreased the likelihood of finishing the race, whereas a higher number of SVP sequences in the second quarter increased the likelihood of finishing the race.

### Runners’ immediate adaptations to the state of vitality loss

Finishers more often experienced an SVL-SVP transition than withdrawers (66.12% against 40%, Table 10). Withdrawers more often experienced two consecutive sequences of SVL than finishers (24.76% against 6.45%). Last, 25.8% of SVL-SVR was observed among finishers against 18.09% among withdrawers. The chi-square test showed a significant difference (χ^2^(2) = 12.21, p = 0.002) in finishers’ and withdrawers’ states at t+1. Note in addition that 90% of withdrawers’ last sequences are in SVL versus 4.76% in finishers.

**Table 10:** Types and frequency of vitality states after a sequence of SVL among finishers and withdrawers.

	SVR		SVP		SVL	
	Finishers	Withdrawers	Finishers	Withdrawers	Finishers	Withdrawers
**%**	25.8	18.09	66.12	40	6.45	24.76

#### Content of the runners’ adaptations

The thematic analysis of the coding showed that both finishers and withdrawers experienced negative physical sensations such as pain, cold and cramps at an immediate level, as identified in the representamens during SVL sequences (Table 11). However, the analysis of involvements showed that finishers attempted to cope with these difficulties by (a) attempting to get back to a preservation state, as shown earlier by the type and frequency of vitality states after a sequence of SVL (see Table 10), and (b) directly and locally reorganizing after an immediate sensation. In contrast, withdrawers (a) more frequently experienced negative physical sensations and (b) had more difficulty getting into a preservation mode and thus tended to remain in SVL. In the same vein, the finishers’ involvements during SVP appeared more focused not only on this experience but also on concerns about maintaining it during the race (Table 12).

**Table 11:** Types of representamens and involvements in finishers and withdrawers in a state of vitality loss.

Finishers		Withdrawers	
Representamens	Involvements	Representamens	Involvements
Gastric pains	Being careful with pace and food	Gastric pains	Hoping it will pass
Muscle cramps and pain	Seeking preservation	Muscle cramps and pain	Trying to hold on, overcoming this state
Fatigue	Having a break	Cold	Trying to warm up
Stress	Trying to relax	Hunger	Should supply
Feeling of difficulty	Not focusing on the performance, just on finishing	Fatigue	Hoping to get better
Foot pain	Adapting the stride	Foot pain	Adapting the stride
Bad mood	Trying to stay positive	Bad mood, negative emotions	Hoping for a better moment to come or thoughts of abandoning
Difficulties of the environmental conditions	Trying to cope and hold on	Difficulties of the environmental conditions	Suffering, thinking of withdrawing

**Table 12:** Types of representamens and involvements in finishers and withdrawers in a state of vitality preservation.

Finishers		Withdrawers	
Representamens	Involvements	Representamens	Involvements
Impression of running at a slow pace	In spite of wanting to accelerate, set oneself to slow down/keep his pace	Concerns about past injuries	Preservation of physical integrity
Good mood	Enjoy each moment	Too much time spent at the aid stations	Careful with food and drink, reserves
Beautiful landscapes	Attempting to finish the race without getting hurt or too exhausted	Medical procedures	Getting healed
Feeling relaxed	Looking for recuperation	Time barriers	Following the pace of another racer
Absence of stress or anxiety	Split the race into smaller stages	People encouraging	Hoping to feel better
Feeling of having a sustainable pace	Should manage the entire race	Being overtaken, others getting ahead	Adapting the stride
Carefulness	Anticipate each potential difficulty	Difficulty to have a regular pace	Avoid getting into physical difficulty

**Fig 4:**
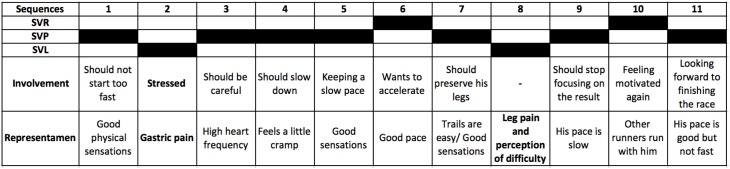
Example of thematic analysis in a finisher.

**Fig 5:**
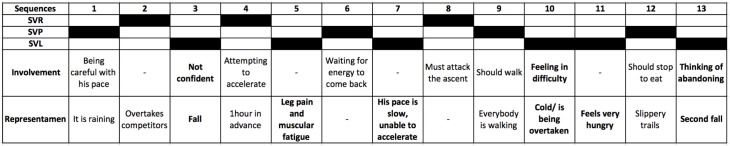
Example of thematic analysis in a withdrawer.

The two examples depicted in Figs 4 and 5 show that both the finishers and withdrawers experienced various difficulties expressed in the representamens that impacted their involvements. The finishers’ involvements indicated an overriding concern with preserving oneself in order to finish the race, specifically by refusing to focus on the performance itself after an experienced SVL (Fig 4). The withdrawers’ involvements indicated various concerns about vitality issues and perceptions of being in difficulty and not being able to stay in preservation (Fig 5).

## Discussion

The aim of this study was to characterize the distribution and temporal organization of the vitality states experienced by runners in a trail race and their adaptations to them, in order to distinguish finishers and withdrawers. Our results showed that the three vitality states emerged in all of them; however, the temporal organization of these experiences suggests that a situated vitality adaptation is a central point in determining whether a runner will finish or withdraw. We must remember that these three vitality states were considered as emerging at the level of the athletes’ experience in relation to what they enacted during their races. They were meaningful parts of the stream of their sense of what happened when they were in the situations and, by performing such actions as accelerating, pacing, or sleeping, they enacted these worlds of feelings, appraisals and thoughts at their pre-reflective level of consciousness [28,29,83]. Having said that, our results clearly showed that finishers and withdrawers did not enact the same world in the race situation, and our in-depth discussion focuses on three points: (a) finishing the race in a vitality preservation state, (b) adapting to a vitality loss state, and (c) the temporal organization of the vitality states.

### Finishing the race in a vitality preservation state

Finishers privileged a world that corresponded to vitality preservation, whereas withdrawers spent less time in preservation and more in a state of vitality loss. These runners thus had to deal with the problem of not only creating a world of preservation but also maintaining it over the entire race in order to finish. We were able to observe how the succession of asymmetrical interactions between runners’ organization and the perturbations emerging from constraints in the race environment distinguished finishers from withdrawers. Finishers were able to preserve their own organization during a meaningful and significant part of the race [9], while withdrawers enacted a new organization in relation to these perturbations that protected them from the troublesome consequences but progressively excluded them from the race.

Our results thus showed that preservation should be the privileged enacted world as it leads to finishing, and switches to loss or revival worlds during the race can have negative impacts on the outcome. Indeed, the finishers might have been able to maintain the enactment of a preservation vitality state because they were able to block the possibility of switching to one of these other worlds. Enacting a world of preservation also meant taking into account the potential risk of overwhelming vitality loss by carefully and continuously monitoring to ensure that a new but ineffective enactment would not emerge. Previous publications [8,9,13] have documented the specific management required for long-distance sports, including recovery time, and sleeping might be one of the performance factors. Hurdiel et al. [8] reported that runners made compromises between racing and resting by taking short naps, which let them complete the race in spite of the cognitive deficits that they observed. During a 4,856-kilometer cycling race, Lahart et al. [13] examined the consequences of sleep deprivation and energy deficiency on four cyclists’ emotions. They found that the cyclists managed less than one hour of continuous sleep per sleep episode: in addition to short sleep duration, inadequate energy intake led to unpleasant emotions and difficulty in regulating them. Moreover, the authors showed that actual sleep and sleep efficiency were better maintained during longer rest periods, highlighting the importance of a race strategy that optimizes the balance between average cycling velocity and sleep time. They suggested that cyclists should: (a) have a plan prepared in advance to ensure sufficient sleep and recovery, (b) develop nutritional strategies to maintain energy intake and thus reduce energy deficits, and (c) anticipate the deleterious effects of sleep deprivation to be able to appropriately respond to unexpected stressors [16]. Our results are in line with these suggestions, which address a broad preservation issue in endurance sports, and expand on them by showing how finishers enacted a world of preservation that also curbed the emergence of the revival option. Here, our thematic analysis of runners’ adaptations suggests that finishers are able to control their propensity to accelerate, even though their immediate feelings might be good, in contrast to withdrawers. Hence, these findings provide further insight into the organization of vitality adaptations and underscore the key role of preservation, a critical factor in finishing ultra-long races. It seems less important to be able to enact a new world after a difficult period than to be able to maintain a preservation state once it is enacted.

Last, our results also showed that the differences between finishers and withdrawers in relation to a preservation strategy are particularly important when the race is shorter. This result appears counterintuitive at first view if we assume that race difficulty is directly linked to distance. However, although no causal link between personality factors and ultra-race participation was found [12], we might interpret this result as indicating that runners in ultra-races are more skillful and pay more attention to their pace and the supply procedures that are vital for finishing the race. This assumption of pace management is in line with the results of Lambert et al. [84], who demonstrated that the faster runners in a 100-kilometer race were able to maintain their initial pace for a longer time compared with the slower runners, who showed greater variation in their pace, which also decreased more rapidly. In contrast, other types of runners, including beginners, might run shorter races but still fall into the traps that would be avoided with sufficient self-awareness. Highly self-aware runners are very careful and pay attention to their physical alerts, fitness and how they are feeling during the run [33]. Interestingly, a study on nutrient intake showed that most amateur runners did not meet their energy intake and nutritional requirements during a mountain marathon [85]. Moreover, we can assume that preservation concerns are stronger when the race is long or known for its difficulty, and the need for preserving oneself during a short race might be underestimated, especially for inexperienced or inadequately trained runners, in line with the finding of a study on effort regulation in rowing between elites and sub-elites [86]. Therefore, runners’ preparation should also be explored and considered as one of the factors of race completion. For example, Krouse et al. [6] investigated female ultra-runners’ training practices and found empirical evidence of self-regulated training practices, such as using their own experience, blogs and websites to complete their training knowledge. Yet, such self-regulated practices might generate incomplete knowledge about the types of preparation needed for these races.

### Adaptation to a vitality loss state

Nevertheless, because withdrawers more frequently enacted longer vitality loss worlds than those of finishers, it is also possible that they became stuck in this state as they were unable to enact an exit. From this perspective, the capacity to enact a new world may be a determinant of outcome. Our thematic analysis of runners’ adaptations confirmed this difference between finishers and withdrawers: Finishers rapidly modified their mode of involvement when this world emerged, whereas withdrawers appeared to focus on their feelings of discomfort. In short, withdrawers contemplated their difficulties, whereas finishers tried to find a better world by enacting local adaptions in response to perturbations. Thus, although both finishers and withdrawers felt vitality loss during the race, as already shown in previous research on ultra-marathons [10,16], finishers enacted new meanings and put aside their difficulties by immediately trying to find solutions to change the world of feelings they were in. Our results therefore also confirm the interpretation that the capacity to immediately enact a new world when feelings of difficulties appear helps to overcome the temptation of race withdrawal. This agrees with the findings of a study on the variation in emotions throughout a multi-stage race regarding the importance of adaptive psychological states [87]: put differently, runners should pay attention to and interpret their emotions, using them as a guide for adapting their activity and thereby ensuring the emergence of a better state for carrying on in the race.

How do withdrawers enact a world of vitality loss? Our results showed that repeated experiences of states of vitality loss were associated with withdrawal, contrasting with the repeated experiences of states of vitality preservation observed for finishers. One interpretation is that the more often an individual enacts a type of world, the easier it becomes to maintain that world, despite any perturbations that may arise. When finishers enacted a more continuous world of preservation, they ensured and reinforced satisfactory levels of relative comfort and economical organization compatible with the race duration [9,33]. In contrast, repeatedly enacting a world of vitality loss reinforced its impact, increased its degree, and progressively led to a pressing need to stop this world from developing further: withdrawers then enacted a new world in an attempt to preserve their long-term viability as ultra-runners.

### Temporal organization of the vitality states

Our results revealed that very early on, in about the first quarter of the race, the differences in the states of preservation and loss between future finishers and withdrawers were significant. This suggests that the race outcome (i.e., finish or withdraw) began to take shape relatively early on and was sensitive to the runners’ initial vitality states. Thus, we can argue that these results can be interpreted as relying on the temporal chain of these states, and particularly on the cumulative effect of the succession of vitality states. When runners began a race and soon after experienced a state of vitality loss, this early experience had a more powerful impact on the following states of vitality loss, which all the runners encountered. Here, a cumulative effect increased the differences we observed between finishers and withdrawers over the course of the race. Each successive state of vitality loss was immediately experienced more powerfully, impacting more negatively on the runners’ overall experience and affecting them more profoundly. In contrast, when finishers, who experienced fewer states of vitality loss, encountered this type of difficulty, they were still feeling sufficiently well and thus had the psychological resources to enact a new state. Experienced states of vitality preservation played the same role but in an opposite direction: the feeling of preservation kept the runners in a state of regular rhythmicity/pacing, and because they had found a comfortable way to run, the kilometers seemed to pass easier and the distance to run seemed less daunting. This phenomenon has already been reported in long-distance walking, during which a cumulative effect of walkers’ positive feelings and emotion increased throughout the duration of the walk [88].

This phenomenon does not rely only on pacing, however, because it is part of a more global experience of running that is made up of many different feelings (e.g., [4,16]). None of the withdrawers found a stable state of preservation, but instead moved from one state to another. This irregularity also explained why they did not find a stable state of relative ease, which would have helped them to continue the race. Instead, the differences with finishers increased throughout the race.

### Methodological issues and limitations

Some methodological aspects of this study should be underlined. The difference in the number of sequences resulting from our coding of the data from the blogs and EIs was not significant, suggesting that we used comparable narratives to document the experience of vitality states. This result is in accordance with Bargh et al. [62] and Jones & Alony [63], who claimed that accounts posted on blogs could be used to obtain valuable data on the personal experience that marked people’s minds. Furthermore, the data extracted from the blogs were considered as primary data, which by definition are not influenced by the researcher’s intervention. The relative anonymity of the blog posts (e.g., use of pseudonyms) is thought to facilitate the expression of what the authors called the “true self” [62]. Therefore, researchers may well be able to access real lived experience. Of course, for this study, we selected specific blog post narratives that rendered this type of analysis possible. Indeed, not all the narratives were adapted for this kind of analysis, because some of them contained inaccurate information, some were humorous narrations, and others were reports about other runners’ activity. A key strength of our data is that we were able to distinguish the states of vitality revival, preservation and loss that were then restored in the chronological logic of the trail runners’ experience. We were also able to document the contents of these vitality states in finishers and withdrawers: thanks to their courses of experience depicted in their narratives, we were able to understand more deeply how they continuously organized their activity.

This perspective is not completely new, but it provides a way to link experiences in other domains of human activity, such as effort, pain, and feelings of ease, in a succession of states, which is innovative in exploring human activity as it is experienced. This research also suggests the need for further reflection on Bruner’s question about the purpose of narrative analysis and whether it should be focused only on specific and singular events in the precise situation in which they occur or whether there are realities common to all narrations [56]. Indeed, in the example of the two races (i.e., the “CCC” and the “Nivolet Revard” races) in which several runners participated (Table 3), we sometimes observed common representamens but, although the runners all had singular experiences, these common representamens did not necessarily have the same impact on their experience.

This study had some methodological consequences. We were able to validate a method of data processing that was less time-consuming than EIs by using high-value narratives directly available on the Internet that portrayed a significant part of trail running experience. Indeed, social media provide a platform on which the sense of the trail running community is important in terms of experience-sharing and race preparation [4].

A study limitation that bears mentioning concerns the qualitative approach: some of our data were collected during post-race interviews, which inherently raises the issue of retrospective recall [89]. However, as noted, we took great care to minimize the effects of retrospective recall by systematically keeping the runners in a re-enactment process. Also, the question of post-race judgment should be addressed: one might argue that the finishers displayed better judgment due to their successful completion of the race. However, our methodological design aimed to reduce this risk because the runners were asked to avoid judgment and to focus on the stream of situated experience. Furthermore, the direct relationship between race outcome and positive/negative judgment about the race is in itself debatable; indeed, some of the finishers were not satisfied with their race performance, whereas some of the withdrawers minimized negative judgments by stating that the decision to quit was the right one [32]. In addition, despite the difficult moments, the withdrawers also mentioned very positive moments with good sensations.

Another limitation has to do with the characterization of the vitality states as discrete. They were presented as a temporal chain, with clear distinctions between them, as this was a necessary step in constructing valid quantitative and qualitative analyses. However, it is quite likely that in a real race vitality states are far less clearly delineated, with states emerging and being experienced more progressively. In this respect, although coding rendered our data clearer, the discretization was also somewhat artificial to highlight the shifts in the runners’ experience through the changes in the representamens and involvements identified in the coding. Yet it is important to note that this procedure is current in research that analyzes the stream of experience using the Experience Sampling Method (e.g., [90]) or the Day Reconstruction Method (e.g., [91]). Also, although we did our best to ensure the accuracy of the experience shifts, we assume that during the race, these changes in the representamens and involvements that the runners were able to report emerged sufficiently strongly in their experience, reducing the fine grain analysis of the shifts.

### Conclusion

This study showed that the notions of (a) seeking preservation, (b) making a good start, (c) delaying the emergence of a state of vitality loss, and (d) being able to exit a state of vitality loss may enrich our understanding of the factors that determine a runner’s ability to finish an extreme race (generally perceived as the capacity for self-surpassing). The notion of self-surpassing might be real when runners remain in a state of vitality loss, especially when they experience suffering without trying to enact a new world. Last, our results suggested that the main challenge for runners is to avoid entering into this state: the more they are able to remain in a state of preservation, the more likely they are to finish.
